# Clinical features, treatment, and survival outcome of primary pulmonary NUT midline carcinoma

**DOI:** 10.1186/s13023-020-01449-x

**Published:** 2020-07-10

**Authors:** Xiao-Hong Xie, Li-Qiang Wang, Yin-Yin Qin, Xin-Qing Lin, Zhan-Hong Xie, Ming Liu, Jie-Xia Zhang, Ming Ouyang, Jun Liu, Ying-Ying Gu, Shi-Yue Li, Cheng-Zhi Zhou

**Affiliations:** 1 Department of Pulmonary and Critical Care Medicine, The First Affiliated Hospital of Guangzhou Medical University, Guangzhou Institute of Respiratory Health, State Key Laboratory of Respiratory Disease, National Clinical Research Center for Respiratory Disease; Guangzhou Medical University, 151 Yanjiang Road, Guangzhou, 510120 China; 2 Department of Thoracic Surgery and Oncology, The First Affiliated Hospital of Guangzhou Medical University, Guangzhou Institute of Respiratory Health, State Key Laboratory of Respiratory Disease, National Clinical Research Center for Respiratory Disease; Guangzhou Medical University, Guangzhou, 510120 China; 3 Department of Respiratory Pathology, The First Affiliated Hospital of Guangzhou Medical University, Guangzhou Institute of Respiratory Health, State Key Laboratory of Respiratory Disease, National Clinical Research Center for Respiratory Disease; Guangzhou Medical University, Guangzhou, 510120 China

**Keywords:** NUT midline carcinoma, Pulmonary, Checkpoint immunotherapy, Survival, Tumour mutational burden

## Abstract

**Objective:**

NUT midline carcinoma (NMC), a rare type of squamous cell carcinoma, is genetically characterised by NUT midline carcinoma family member 1 (NUTM1) gene rearrangement. NMC can arise from the lungs; however, there is no standard for the management of primary pulmonary NMC. This study aimed to confirm the clinical features and report the treatments, especially with immune checkpoint inhibitors (ICIs), and outcomes of patients with primary pulmonary NMC.

**Methods:**

A retrospective review of patients with primary pulmonary NMC was performed in the First Affiliated Hospital of Guangzhou Medical University between January 2015 and December 2018. Clinical manifestations as well as radiographic and pathological findings were recorded. Whole-exome sequencing (WES), a predictor for ICI response, was used to determine the tumour mutational burden (TMB). Treatments, especially by immune checkpoint blockade, and patient survival were analysed.

**Results:**

Seven patients with primary pulmonary mass (four men and three women) with a mean age of 42 years (range, 23–74) who were diagnosed with NMC according to NUT immunohistochemistry staining were included for analysis. One patient had a rare fusion of CHRM5-NUTM1 by tumour sequencing. A wide range of TMB (1.75–73.81 mutations/Mbp) was observed. The initial treatments included chemotherapy (5/7, 71.4%), surgery (1/7, 14.3%), and radiotherapy (1/7, 14.3%). Five patients (5/7, 71.4%) received ICIs (programmed cell death protein 1 [PD1]/programmed cell death ligand 1 [PDL1] monoclonal antibody) as second- or higher-line treatments. The median overall survival (OS) was 4.1 months (range, 1.5–26.7 months).

**Conclusions:**

Patients with primary pulmonary NMC have a poor prognosis and chemotherapy is often preferred. Checkpoint immunotherapy is a good option as the second- or higher-line treatment. TMB seems to be not associated with OS.

## Introduction

NUT midline carcinoma (NMC) is a rare, highly aggressive, and poorly differentiated squamous cell carcinoma genetically characterised by a NUT midline carcinoma family member 1 (NUTM1) gene rearrangement on chromosomal 15q14. The main fusion partner is bromodomain-containing protein 4 (BRD4) on chromosome 19p13.1, which results in the BRD4-NUT oncogene driven by the BRD4 promoter. The less common translocation partners include BRD3 and nuclear receptor binding SET domain protein 3 (NSD3) [1–3]. The BRD-NUT fusion protein causes epigenetic reprogramming and loss of cell differentiation, which results in the relatively consistent phenotype and function of NMC [4]. NMC arises from many organs, mainly midline organs such as the head, neck, and thorax, and usually has widespread metastases when diagnosed [5]. Most patients have advanced stages of the disease and progress rapidly to death, with a median overall survival (OS) of 6.7 months and 2-year progression-free survival (PFS) rate of 9% [6].

Analysis of the largest cohort of NMC patients (141 patients) showed that 50% had primary tumours of thoracic origin; however, the proportion of primary lung NMC was unknown [7]. Another cohort of 138 NMC cases reported a lung incidence of 34% [8]. A large case series of primary lung NMC summarizing detailed clinical and pathological courses reported only one (0.6%) case among 166 lung biopsy specimens lacking glandular differentiation [9].

The histological morphology of NMC is unique but not specific. Abrupt squamous differentiation helps to distinguish NMC from undifferentiated tumours; however, it is also present in some squamous cell carcinoma [5, 10, 11]. Immunohistochemistry (IHC) using a monoclonal antibody to NUT, with extremely high specificity (100%) and sensitivity (87%), can be a cost-effective and rapid diagnosis approach [12]. In addition, IHC often shows expression of keratin and p40/p63 and, less often, of thyroid transcription factor 1 (TTF-1), synaptophysin, and CD56 [9]. Other diagnosis approaches for detecting the gene fusion (BRD4-NUT) include fluorescence in situ hybridization (FISH), reverse transcriptase-polymerase chain reaction (RT-PCR), and next-generation sequencing (NGS) [1, 13–15]. Whole-exome sequencing (WES) is an NGS-based approach that can be used to measure tumour mutational burden (TMB) and gene mutations in lung cancer as well as BRD4-NUT gene fusion [16–18].

There is no standard management for primary lung NMC. Chemotherapy seems to be ineffective [6]. Surgical resection and initial radiotherapy (RT) may prolong patient PFS and OS [6, 19]. However, surgical inaccessibility often leads to dismal PFS and OS in patients with advanced disease. Bromodomain and extra-terminal motif (BET) inhibitors (BETi) competitively inhibit the binding of BRD4 to chromatin NUTM1, which depletes BRD4-NUT from chromatin and induces rapid growth arrest and terminal differentiation of NMC cells in vivo and in vitro. NMC patients with multiple-line treatment failure still benefit from BETi, suggesting that targeted inhibition of BRD-NUT oncogenes is a promising treatment [5, 20, 21]. Unfortunately, BETi is not available in China. Herein, we retrospectively analysed the clinicopathologic characteristics and imaging of seven patients with primary pulmonary NMC identified in the pathology records between January 2015 and December 2018. WES was performed on available specimens to determine the TMB, gene mutations, and rare fusions of lung NMC. The survival outcomes of concurrent chemoradiotherapy and immunotherapy were also analysed.

## Patients and methods

### Patients

We searched the pathological records of the First Affiliated Hospital of Guangzhou Medical University for cases of primary lung NUT cancer diagnosed by IHC between January 2015 and December 2018 using the keywords “middle line cancer” and “NUT carcinoma”. Tumours considered to arise at extrapulmonary sites were excluded. Seven cases diagnosed with primary pulmonary NMC were identified at our clinical centre. Paraffin-embedded tissues for all cases were available for FISH. The detailed clinical and pathological data for all cases were obtained from their electronic medical records.

### Imaging examinations

Two independent radiologists reviewed the radiological findings, including images and reports at the time of diagnosis. Chest digital radiography (DR) and computed tomography (CT) scans of the patients were available. Additional imaging studies, including abdominal CT, bone scintigraphy, 18F fluorodeoxyglucose (FDG) positron emission tomography (PET)/CT, head CT, and brain magnetic resonance (MR) imaging, were also reviewed if available. Chest CT scans were mainly focused on assessing the location and characteristics of the lung lesion and related pulmonary parenchymal abnormalities, lymphadenopathy, pleural and skeletal abnormalities, and involvement of the contralateral lung. PET/CT and other imaging studies were reviewed to identify the involvement of extra-thoracic sites. Subsequent radiological results were used to assess therapeutic efficacy and metastatic sites.

### Immunohistochemistry (IHC)

IHC analysis of the NMC tissues was performed on 4-μm thick formalin-fixed paraffin-embedded (FFPE) sections. Slides were heat-mediated and antigen-repaired in citrate buffers (pH 6) and then incubated with primary rabbit monoclonal anti-NUT antibody (ab122649) (Abeam, Cambridge, MA) and visualised by Bond Polymer Refine Detection (Leica Microsystems, Buffalo Grove, IL). Two or more independent pathologists interpreted the final visualised NUT IHC staining. Intense spotted nuclear staining in the nuclei greater than 50% was considered interpretable.

### Fluorescence in situ hybridization (FISH)

A two-colour separation FISH assay was performed on 4-μm thick FFPE tissue sections to confirm chromosomal disruption of the NUTM1 gene. The NUTM1 gene on 15q14 was probed separately using RH54191 (211 KB, green) and SHGC-110339 (306 KB, red) (Ambiping [LBP], Guangzhou, China). Positive slides were defined as those with greater than 80% hybridization efficiency in the four regions (200 cells/area) of the FFPE sections.

### Next-generation sequencing (NGS)

FFPE DNA extraction was performed using a QIAamp DNA Mini Kit (Qiagen, Hilden, German) and quantified by Qubit 2.0 Fluorometer (Invitrogen, Carlsbad, CA, USA). DNA libraries were constructed using the SureSelectXT Human All Exon V5 (Agilent Laboratories, Santa-Clara, CA) and the SureSelectXT reagent kit (Agilent Laboratories, Santa-Clara, CA). DNA fragmentation, purification, and in-solution hybrid selection were performed according to the protocol from WuXi NextCODE Co., Ltd. (Shanghai, China). The DNA libraries were subjected to quality inspection by agarose gel electrophoresis, Qubit quantification, and 2100 Bioanalyzer fragment length determination. Sequencing was performed on a HiSeq Sequencing System (Illumina Inc., San Diego, CA). Data analysis was performed according to Illumina’s standard protocol. For patients without specimens for WES, we reviewed large panel sequencing (aka, targeted region sequencing [TRS]) (Burning Rock Biotech, Guangzhou, China) reports containing 295 target genes and small panel sequencing (Beijing Genomics Institute, Beijing, China) reports containing 11 target genes (epidermal growth factor receptor [*EGFR*]; KRAS proto-oncogene, GTPase [*KRAS*]; B-Raf proto-oncogene, serine/threonine kinase [*BRAF*]; Erb-B2 receptor tyrosine kinase 2 [*ERBB2*]; discoidin domain receptor tyrosine kinase 2 [*DDR2*]; MET proto-oncogene, receptor tyrosine kinase [*MET*]; phosphatidylinositol-4,5-bisphosphate 3-kinase catalytic subunit alpha [*PIK3CA*], NRAS proto-oncogene, GTPase [*NRAS*]; ALK receptor tyrosine kinase [*ALK*], ROS proto-oncogene 1, receptor tyrosine kinase [*ROS1*]; and ret. proto-oncogene [*RET*]) at the time of their initial diagnosis.

## Results

### Clinical features of NMC patients of the lung

This study evaluated seven patients (four men and three women) with a mean age of 42 years (range, 23–74). The median follow-up duration from symptom onset to the last oncology visit was 4.1 months (range, 1.5–26.7 months). The clinical features of the patients are described in Table 1. The predominant clinical manifestations were cough (*n* = 7), dyspnoea (*n* = 4), other symptoms including chest pain and fever (*n* = 2), and haemoptysis (*n* = 1). Four cases (all males) had a history of smoking. All patients were previously healthy except for Case 1, with a history of chondrosarcoma. All patients had advanced-stage disease at the time of diagnosis and had Eastern Cooperative Oncology Group (ECOG) performance status (PS) scores between 1 and 2. Epstein–Barr virus (EBV) tests were negative in all patients.

**Table 1:** Clinical features of seven patients with primary pulmonary NUT midline carcinoma (NMC)

Patients	Age (years)	Sex	Symptoms (duration; months)	TNM staging	Smoking history	History of malignant tumour	ECOG PS	EBV	HPV
1	23	M	Cough, fever, chest pain (1)	pT3N2M1b (IVa)	180-pack years	chondrosarcoma	2	–	NA
2	53	M	Cough (3)	cT3N3M1b (IVa)	240-pack years	NA	1	–	NA
3	30	F	Cough (2), gasping (0.5)	cT4N3M1b (IVa)	Never	NA	2	–	NA
4	25	M	Cough, chest pain, fever, dyspnoea (1)	cT4N2M1a (IVa)	90-pack years	NA	2	–	NA
5	74	M	Cough, haemoptysis (2)	cT3N3M1a (IVa)	360-pack years	NA	1	–	NA
6	58	F	Cough, gasping (0.5)	cT4N3M0 (IIIc)	Never	NA	1	–	NA
7	31	F	Cough (3), dyspnoea (2)	cT4N1M0 (IIIa)	Never	NA	2	–	NA

- negative, *NA* data not available

### Imaging examination findings

The radiographic features of the seven patients are detailed in Table 2. Chest DR mainly showed irregular bands with unclear boundaries, shadows of uniform density with blurred boundaries, and enlarged hilar lymph nodes. Chest plain CT scans revealed irregular low-density masses, whereas enhanced CT scans showed uneven enhancement of the masses. The lesions were large, with a longest diameter of 5–12.7 cm and were mostly located in the right lung. Four of the five of primary pulmonary lesions were centrally located. Lesions invaded the ipsilateral lung and fused with the ipsilateral and mediastinal lymph nodes. Two patients had small or limited pleural effusion. Patients with primary tracheal lesions showed no disease in the thoracic cavity or lungs except for hilar lymphadenopathy. Regional infiltrating lymph nodes were detectable in all cases. Strong FDG-avidity with SUV_max_ of 10.6–18.6 was observed in 3 patients using PET-CT,. Case 5 had IHC positivity in the left mass, with a different SUV in the right lung, was considered to be metastatic. PET-CT showed bone metastases in two patients at the time of diagnosis and CT showed bone metastases in Case 6 before immunotherapy. All cases were negative for brain metastases (five by brain MR and two by brain CT).

**Table 2:** Imaging findings

Patients	Site	Size (cm)	Lung	Pleura	Contralateral lung involvement	Lymphadenopathy	Extrathoracic sites	SUV
1	RUL, Central	5.5 × 4.1	Post-obstructive atelectasis	Large effusion, Pleural nodules	Small right-sided effusion	Hilar(L), Mediastinal(B)	Bone	10.6
2	RUL, Central	5.4 × 3.7	Post-obstructive atelectasis	Small effusion	None	Supraclavicular(R), Hilar(R), Mediastinal(R)	Bone Adrenal gland	18.6
3	RLL, Central	4.7 × 4.7 × 4.7	Post-obstructive atelectasis	Medium pleural effusion	Small left-sided effusion	Supraclavicular(B), Hilar(B), Axillary(B), Mediastinal(B)	Bone	NA
4	RLL, Central	10 × 6.4 × 12.7	Intrapulmonary metastasis, Pulmonary vascular invasion	Large effusion, Pleural nodules	None	Hilar(R), Mediastinal(R)	Pericardium, back(R)	NA
5	LLL, RUL, Peripheral	2.8 × 2.0 5.3 × 4.8	None	None	Lung	Supraclavicular(R), Hilar(L), Mediastinal(L)	None	13.6 18.4
6	OT	4 cm long	None	None	None	Posterior tracheal, Hilar(R),Mediastinal(B)	Oesophagus	NA
7	LT	3.0 × 3.1 × 3.1	None	None	None	Mediastinal(B)	None	NA

*RLL* right lower lobe, *RML* right middle lobe, *RUL* right upper lobe, *LUL* left upper lobe, *LLL* left lower lobe, *LT* lower trachea, *OT* origin of trachea, *NA* data not available, *B* bilateral, *R* right, *L* left, *SUV* standard uptake value

### Histological features

The pathological characteristics are shown in Table 3. Two cases were diagnosed with mucinous epithelial carcinoma and squamous cell carcinoma, respectively, and were re-diagnosed as NMC after IHC for NUT. Surgical specimens from Case 1 showed cancerous tissue metastasis in the lungs, bronchus infiltrates, and visceral and parietal pleura involvement. Cytological morphological features included cancer cells with round, elliptical, or irregular nuclei in a nested arrangement. Nuclear fission and prominent nucleoli were observed in the nucleus. The cytoplasm was abundant and red- or light-stained. One case showed small blue, round cells with a partially extruded deformation and an irregular nucleus accompanied by significant neutrophil infiltration, hyperplastic fibrous tissue, and dilated blood vessels. In Case 6, keratinised bead formation was observed in poorly differentiated tumours. IHC showed that all specimens were positive for NUT and CK, of which six were positive for P63/P40, five were positive for Ki-67 (40 to 70%), and two were positive for TTF-1 and CD5/6. Most patients were negative for Napsin A and CgA/Syn. Among the seven patients whose specimens were analysed by FISH, those from Case 2 could not be stained and only Case 7 showed positive results.

**Table 3:** Pathological characteristics

Patients	NUT	CK	P63	P40	CD5/6	TTF1	NapsinA	CgA/Syn	Ki-67	Suggested diagnosis	Modified diagnosis	FISH
1	+	+	+	ND	+	–	–	–	50%+	mucinous epithelial carcinoma	NMC	–
2	+	+	+	+	–	+	–	–	70%+	NMC	–	
3	+	+	+	ND	–	–	ND	–	40%+	squamous cell carcinoma	NMC	–
4	+	+	ND	–	ND	–	ND	ND	70%+	NMC	–	–
5	+	+	+(focal)	+(focal)	–	–	–	–	ND	NMC	–	–
6	+	+	+	+	ND	–	ND	ND	ND	NMC	–	–
7	+	+(focal)	+	+	+	+	–	–	40%+	NMC	–	+

*ND* not done

### NGS

**Fig. 1:**
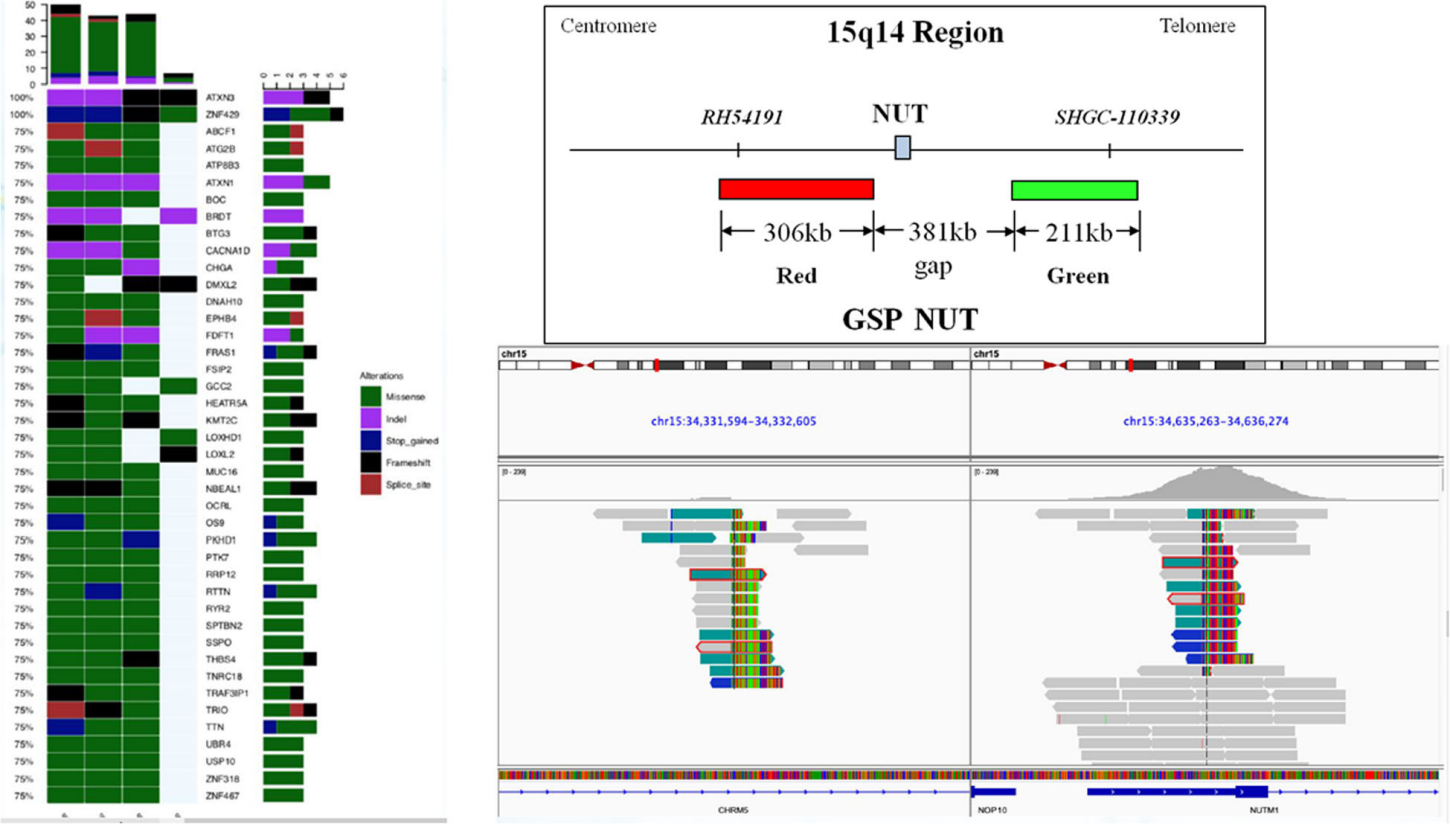
The tumour sequencing results

The tumour sequencing results are summarised in Table 4 and shown in Fig. 1. A total of five FFPE specimens and two blood samples from five patients were submitted for WES. In the NGS-QC assessment of tissue specimens, only Case 2 did not meet the criteria for conducting WES. Among patient samples submitted for WES, the DNA degradation of all blood samples and the specimen from Case 7 were grade A and the remaining samples were grade B. Although other specimens showed a higher TMB, no fusion partner of NUTM1 was found. Only Case 7 showed a lower TMB and a rare CHRM5-NUTM1 (intron1-5UTR, 12.47%) fusion. Forty-two genes were detected in at least three samples (Fig. 1), and ataxin 3 (*ATXN3*) and zinc finger protein 429 [*ZNF429*] mutations were detected in all samples. Functional enrichment analysis (Go/KEGG analysis) of the genes mutated at high frequency did not reveal significantly enriched cancer-associated pathways. Among the patients without WES, only Case 2 had a TRS report at the time of initial diagnosis. The TRS report of Case 5 showed high TMB and an exon 6 nonsense mutation in phosphatase and tensin homolog (*PTEN*) with an allele fraction of 70.64%. The small-panel TRS reports showed an *EGFR* exon 19 deletion in Case 2 and a phosphoinositide-3-kinase regulatory subunit 1 (*PIK3R1*) p.I29L mutation with an allele fraction of 70.64%.

**Table 4:** Tumour sequencing results

Patients	Tissue	WBC	DNA degradation	OD value (ng/μL)	Qubit (ng/μL)	TMB	NUTM1-fusion	Mutations
1	Primary lung tissue	No	B	226	88	11.55	No	ATXN3 ZNF429
2	Primary lung tissue	No	C	9.533	1.4	unqualified	NA	EGFR exon 19 del^a^
4	Primary lung tissue	No	B	9.383	1.9	58.79	No	ATXN3 ZNF429
5	Primary lung tissue	Yes	NA	NA	NA	High^b^	No	PTEN^b^
6	Primary lung tissue	Yes	B	7.884	1.7	73.81	No	ATXN3 ZNF429
7	Primary lung tissue	Yes	A	306.3	145.5	1.75	CHRM5-NUTM1	ATXN3 ZNF429 PIK3R1 p.I29L^a^

^a^Reported from small panel

^b^Reported from large panel

### Treatments and outcomes

**Fig. 2:**
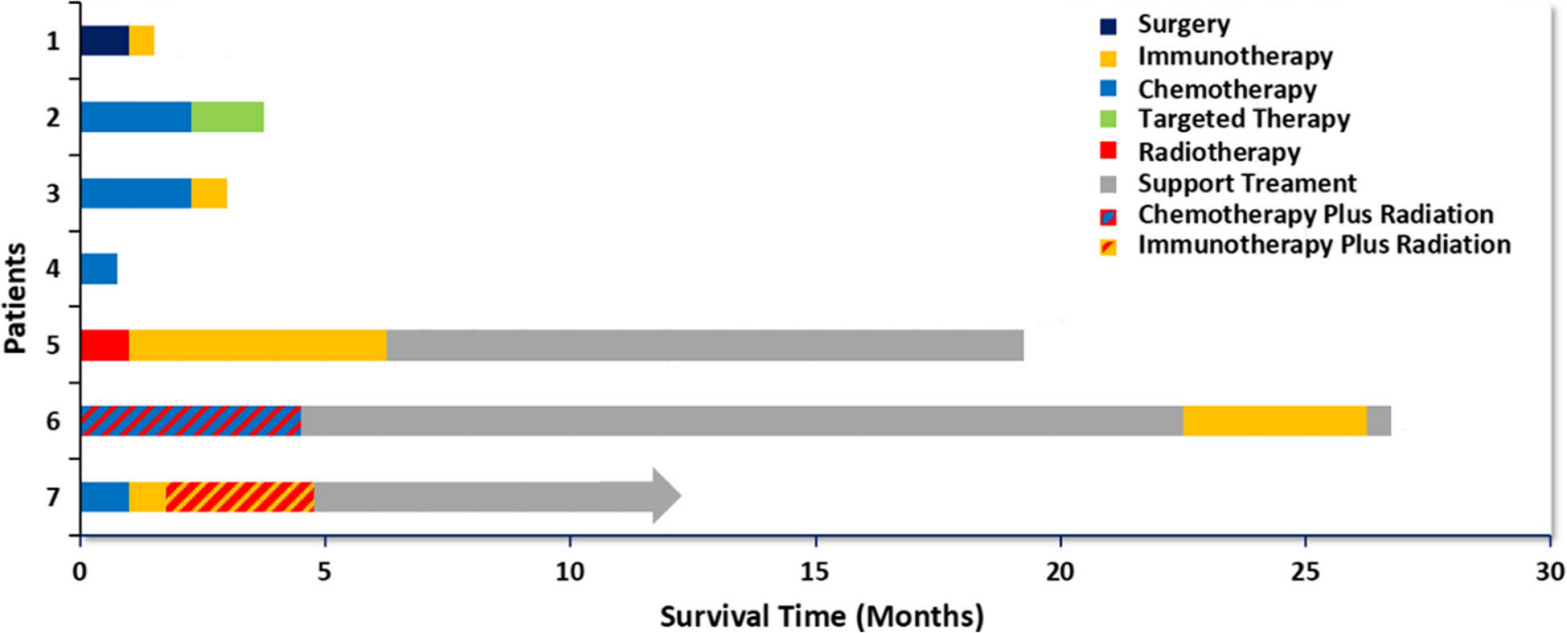
Treatments and survival outcomes of the seven patients

Treatments and survival outcomes of the seven patients are shown both in Table 5 and Fig. 2.After confirmed diagnosis, 5 patients received chemotherapy, and another two patients received surgery and radiotherapy, respectively. A total of 5 patients received immune checkpoint inhibitors (anti-PD1/PDL1 monoclonal antibody) as the second or above-line treatments. The median OS was 4.1 months (range, 1.5–26.7 months). Case 1 was administered atezolizumab after surgery, but died of severe postoperative complications. Cases 3 and 5 were administered nivolumab and pembrolizumab, respectively. Case 5 had clinical response to PD-1 inhibitor, with an OS of 19.5 months, while case 3 seemed to fail to benefit from immunotherapy, with just an OS of 3 months. Another 2 patients, case 6 and case 7 had long OS of more than 1 year (26.7 and 12 months, respectively). Interestingly, patients with tracheal masses (case 6 and case 7) had long PFS when receiving immunotherapy as second- or third-line treatments. Case 6 obtained a partial remission for 18 months after 4 months of concurrent chemoradiotherapy, but Case 7 did not benefit from chemotherapy. Moreover, they had much longer OS than the patients with pulmonary masses. Except case 2,the median OS (mOS) in patients with pulmonary masses was 2.75 months (range 1.5–4.1 months).

**Table 5:** Treatments and outcomes

Patients	Treatment1	PFS1	Treatment2	PFS2	Treatment3	PFS3	Treatment 4	PFS3	Treatment5	PFS4	Outcomes	OS
1	Surgery	1 m	Atezo	2w	–	–	–	–	–	–	dead	1.5 m
2	TP	3 × 21d	Gefitinib	2w	Apatinib	1 m	–	–	–	–	dead	4.1 m
3	TP	2 × 21d	T	1 × 21d	T + Nivo	1 × 21d	–	–	–	–	dead	3 m
4	TP	21d									dead	1.5 m
5	RT	1 m	Pembro	7 × 21d(5 m)	Support care	13 m	–	–	–	–	dead	19.5 m
6	Cet + DP + CCR	6 × 21d	Support care	18 m	Pembro+Cet	4 × 21d	Pembro+ oxaliplatin	1 × 21d	Support care	–	dead	26.7 m
7	DP + Cet	2 × 21d	G + T + Nivo Pembro+Nivo+GT	2w 2 × 14d	Nivo+Iri + P	21d	Pembro+CBP + R	3 m	Support care	–	Alive	12 m+

*TP* paclitaxel-albumin(T)+carboplatin, *DP* docetaxel(D) + platinum, *Cet* cetuximab, *Nivo* nivotuzumab, *CCR* concurrent chest radiotherapy(RT), *Atezo* atezolizumab, *Pembro* pembrolizumab, *Iri* irinotecan

## Discussion

Descriptions of primary pulmonary NMC are rare and have only been reported in a few case reports and case series; thus, it is necessary to summarise the clinical features and treatment of this entity. Herein, we summarised the clinical and pathological processes of seven patients with primary pulmonary NMC and explored the efficacy of different treatment options. Furthermore, WES was used to explore the TMB, gene fusions, and potential coexisting mutations. We also explored the relationship between TMB and immunotherapy efficacy.

The median ages of diagnosis for patients with lung cancer and NMC overall were 70 and 24 years, respectively [7, 22], while the median age of this group of patients was 31 years (mean 42 years). All patients were diagnosed in an advanced stage with a poor PS score. Before diagnosis, all patients complained of a cough lasting for more than a month and some patients had difficulty breathing, chest pain, fever, or haemoptysis. Four patients had a significant smoking history while three patients had no or negligible tobacco exposure. Consistent with previous studies, all patients had no EBV exposure, suggesting that EBV was not associated with NMC [5]. No longer restricted to paediatric patients and midline structures, with the development of diagnostic techniques, this entity has been reported in patients of all ages (0–81.7 years) and in non-midline structures [19]. Therefore, regardless of the primary lung mass in young or elderly patients, even those without a smoking history, NMC should always be included in the differential diagnosis of malignant lung tumours when the clinical course appears to be aggressive.

Similar to the resulted reported by Lynette, the imaging results lacked specificity but had some distinct features [9]. Chest CT showed a large primary pulmonary mass (diameter ≥ 5 cm) fused with the hilar and mediastinal lymph nodes, with obstructive atelectasis and ipsilateral pleural involvement, while the contralateral lungs were essentially unaffected. Furthermore, local invasion involved the oesophagus and back, with distant metastases mainly involving the adrenal glands and bone. In our study, bone metastases were the most common (3/7) extrathoracic metastases assessed by CT and PET/CT. Brain metastases were most common site in lung cancer compared to other cancers with cumulative 1-years incidence rates of 3.0% in patients with Stage III non-small cell lung cancer (NSCLC) and 10.8% in patients with Stage IV NSCLC [23, 24]. Brain metastases from lung NMC have not been reported and it is often believed that the tumours progress too rapidly to cause brain metastasis. Consistent with previous studies, no brain metastases were observed by brain CT or MR, including in the three patients who survived for more than 1 year.

The cytomorphology of NMC originating in the lungs and other sites has been extensively described [5, 9, 25]. The histological manifestations of this entity can be confused with other high-grade lung malignancies and lead to the misdiagnosis of NMC [9]. In our series, two patients were initially diagnosed with mucinous epithelial carcinoma and squamous cell carcinoma before undergoing NUT IHC. Therefore, the possibility of NMC lung cancer cannot be ruled out in cases manifesting as other poorly differentiated tumours. Consistent with previous studies, cases with pulmonary NMC mainly expressed keratin, P63, and/or P40 in IHC, which suggests that NMC cells are derived from squamous cell carcinoma [9, 25, 26]. Two patients (Cases 2 and 7) were positive for TTF-1 expression, which favoured the diagnosis of lung adenocarcinoma [27]. The significance of TTF-1 expression in this entity is unclear.

Four patients in our series underwent WES due to fewer specimens and unqualified quality assessment. Among them, only Case 7 (with the lowest TMB) was confirmed to have a rare fusion of CHRM5-NUTM1 (intron1-5UTR, 12.47%). We did not identify a NUTM1 fusion partner in the remaining three patients with high TMB. This may be caused by the significant limitations of WES as it does not capture intronic regions where fusion breakpoints often occur [28]. In addition, samples from three patients with high TMB had significant DNA degradation, which may have led to false-positive mutations, resulting in significantly higher TMB. *ATXN3* and *ZNF429* are co-mutated genes detected in all samples but were not found to be associated with cancer pathways in functional enrichment analysis. In short, while WES can be used to detect the fusion partners of *NUTM1*, it has limitations and specimen preservation significantly impacts TMB detection.

The reported mOS of the largest series of NMCs originating from all sites was 6.7 months [6]. In our series, except Case 5, patients with lung masses died of the disease within 5 months despite receiving multiple treatments. The mOS of patients with lung masses was only 2.75 months, while that of patients with tracheal masses was more than 1 year. These findings suggest that NMC patients with tracheal masses have a longer OS than that in patients with lung masses.

There is currently no standard protocol for the treatment of primary pulmonary NMC, and exploration of effective treatment options is urgently needed. Although surgery is an effectivein early-stage primary pulmonary NMC, it may not prolong survival in advanced disease. In our series, Case 1 underwent surgery but eventually died of surgical complications. The patients can benefit from BETi even after multi-line treatments, but the accessibility of the drug limits its application [21]. Therefore, chemotherapy or RT is inevitable in patients with advanced disease. Consistent with previous studies, chemotherapy did not significantly prolong PFS and OS in the patients in this study, whereas concurrent chemoradiotherapy significantly prolonged the OS in Case 6 [6]. Therefore, concurrent chemoradiotherapy may play an important role in prolonging patient survival.

Rapid squamous differentiation caused by knockdown of NUT fusion oncogenes in NMC cell lines suggests that NMCs are derived from squamous cells [29]. PD-1/PD-L1 inhibitors prolong OS in NSCLC patients and, therefore, may be effective against primary pulmonary NMC [30]. Immunotherapy is more effective in patients with high TMB and/or PD-L1 expression [31, 32]. In the present study, immunotherapy was received by five patients. In the patients with pulmonary masses, only Case 5 received RT as a first-line treatment and showed significant efficacy, with an OS of 19.5 months. In contrast, Case 3 received chemotherapy as a first-line treatment but had a very poor prognosis. Although Case 1 had high TMB and received immunotherapy, he died of severe postoperative complications 1 month after surgery. The high TMB may better explain the long survival of Case 2, who was administered immunotherapy. However, given the small number of cases and the long survival time of patients with primary tracheal tumours (case 6 and case 7), it is difficult to prove that high TMB predicts better immunotherapy outcome in these patients. RT plus immunotherapy may prolong survival in NMC patients. After receiving RT, Case 5 was administered with pembrolizumab and obtained prolonged OS of 19.5 months. RT may activate noninflamed tumour toward a more inflamed tumour microenvironment (TME) [33]. Moreover, RT can lead to increased tumor antigen release, improved antigen presentation, upregulated PD1 expression and increased CD8/CD4 ratio in TME. Pembrolizumab by blocking PD1 further enhances immune attack on tumour. Despite benefit of RT in prolonging OS, radiation dose-escalation should be performed cautiously because of the potentially serious complications. Moderate-dose delivering and dose optimization are suggested to improve RT satety [34].

Our study detailed the clinical and pathological features of lung NC and treatment experience and explored the diagnosis of WES in NC. Our case series summarised the largest number of patients administered checkpoint immunotherapy in lung NC and explored the efficacy of immunotherapy. The results suggested that immunotherapy can benefit patients with these deadly tumours. However, our study has certain limitations. This retrospective study of rare lung NC included a limited number of patients. The initial diagnosis of all patients was based on positive NUT-IHC results and the commercial probe did not detect the presence of NUT rupture in all patients. Based on these limitations, the results of exploration of immunotherapy in lung NC remain controversial. More studies on lung NC are needed to confirm our findings.

## Conclusions

The results of this study showed the characteristic diagnostic features of patients with primary pulmonary NMC, including poor PS score at initial diagnosis; large unilateral lesions involving the lung, pleural, and mediastinal lymph nodes; and of keratin, p63, or p40 expression in the primary tumour. Smoking and viral infections were not significantly associated with disease. Tumours with these clinicopathological and radiographic characteristics should be screened by NUT IHC and further assessment of fusion partners by FISH and WES can be used to identify rare fusion partners. Although NMC of the lung is rapidly lethal, patients with tracheal masses survive significantly longer than those with lung masses. NMC is a disease with dismal prognosis where conventional treatments of surgery and chemotherapy seem to fail though some responses and prolongation of OS were seen in patients receiving immunotherapy and RT.

## Data Availability

Identifying patient information must remain confidential; however, additional data may be available upon reasonable request at the discretion of the corresponding author.

## Abbreviations

ALK: ALK receptor tyrosine kinase
ATXN3: Ataxin 3
BETi: Bromodomain and extra-terminal motif inhibitors
BRAF: B-Raf proto-oncogene, serine/threonine kinase
BRD4: Bromodomain-containing protein 4
CT: Computed tomography
DDR2: Discoidin domain receptor tyrosine kinase 2
DR: Digital radiography
EBV: Epstein–Barr virus
ECOG: Eastern Cooperative Oncology Group
EGFR: Epidermal growth factor receptor
ERBB2: Erb-B2 receptor tyrosine kinase 2
FDG: 18F fluorodeoxyglucose
FFPE: Formalin-fixed paraffin-embedded
FISH: Fluorescence in situ hybridization
ICIs: Immune checkpoint inhibitors
IHC: Immunohistochemistry
KRAS: KRAS proto-oncogene, GTPase
MET: MET proto-oncogene, receptor tyrosine kinase
MR: Magnetic resonance
NMC: NUT midline carcinoma
NGS: Next-generation sequencing
NRAS: NRAS proto-oncogene, GTPase
NSCLC: Non-small cell lung cancer
NSD3: Nuclear receptor binding SET domain protein 3
NUTM1: NUT midline carcinoma family member 1
OS: Overall survival
PD1: Programmed cell death protein 1
PDL1: Programmed cell death ligand 1
PET: Positron emission tomography
PFS: Progression-free survival
PIK3CA: Phosphatidylinositol-4,5-bisphosphate 3-kinase catalytic subunit alpha
PIK3R1: Phosphoinositide-3-kinase regulatory subunit 1
PS: Performance status
PTEN: Phosphatase and tensin homolog
RET: Ret. proto-oncogene
ROS1: ROS proto-oncogene 1, receptor tyrosine kinase
RT: Radiotherapy
RT-PCR: Reverse transcriptase-polymerase chain reaction
TMB: Tumour mutational burden
TME: Tumour microenvironment
TRS: Targeted region sequencing
TTF-1: Thyroid transcription factor 1
WES: Whole-exome sequencing
ZNF429: Zinc finger protein 429
